# Controlled preparation of PAMS hollow core microcapsules with high uniformity and its application in the production of GDP fuel capsules for ICF engineering

**DOI:** 10.1016/j.fmre.2022.01.004

**Published:** 2022-01-29

**Authors:** Qiang Chen, Meifang Liu, Xiangdong Liu, Bo Li, Yongping Chen

**Affiliations:** a Laser Fusion Research Center, China Academy of Engineering Physics, Mianyang 621900, China; b Key Laboratory of Energy Thermal Conversion and Control of Ministry of Education, School of Energy and Environment, Southeast University, Nanjing 210096, China

**Keywords:** Inertial confinement fusion engineering, Mass transfer regulation, PAMS HCMs, Spherical uniformity, Surface finish

## Abstract

Uniform poly-α-methylstyrene (PAMS) hollow core microcapsules (HCMs) are widely used as templates to fabricate glow discharge polymer (GDP) fuel capsules, which are fundamental devices for inertial confinement fusion (ICF) engineering. The sphericity and surface finish uniformity of PAMS HCMs are critical for achieving high-quality GDP fuel capsules. In this work, millimeter-scale PAMS HCMs were fabricated by a microencapsulation technique. The sphericity and surface finish uniformity were concurrently improved using di-t-butyl peroxide (DTBP). The mechanisms of these effects were also experimentally and theoretically investigated. The results show that DTBP distributes at the O-W2 interface of W1/O/W2 compound droplets, which resists the diffusion of molecules through the O-W2 interface bidirectionally. The resisted diffusion of H_2_O molecules into the O phase eliminates PAMS HCM surface defects. Additionally, the resistance of fluorobenzene (FB) molecules from diffusing from the O phase into the W2 phase can effectively extend the solidification of W1/O/W2 compound droplets and thus improve the spherical uniformity of the HCMs. Using these improved PAMS HCMs, GDP fuel capsules meeting the stringent requirements for ICF engineering are prepared, and the quality of which is beyond the National Ignition Facility standard.

## Graphical abstract

**Figure:**
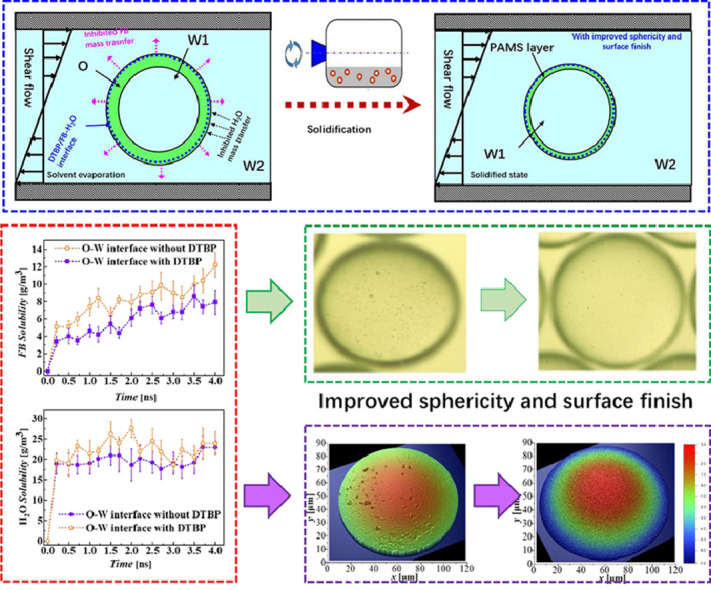

## 1 Introduction

**Fig. 1:**
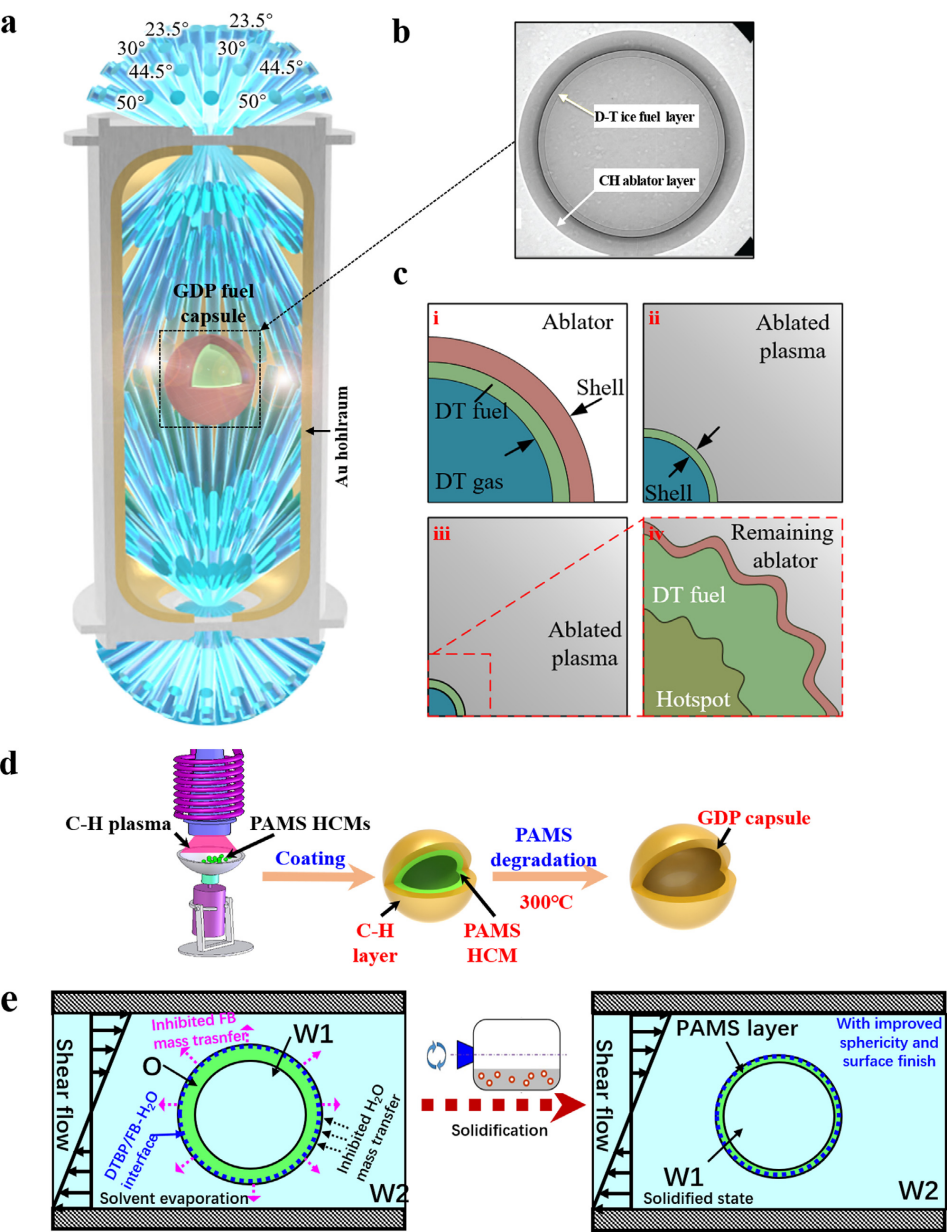
**Schematics of indirectly driven, inertial confined fusion (ICF) targets:** (a) Cross section of the Au hohlraum and GDP fuel capsule with representative incident laser beams; (b) X-ray image of the actual GDP fuel capsule for ICF; (c) implosion process and the induced interface instability; (d) fabrication of the GDP capsule; (e) improvement of PAMS HCMs with the designed bidirectionally inhibited-mass-transfer interface.

As the most promising controlled thermonuclear fusion, inertial confinement fusion (ICF), which aims to generate clean and economic energy for the future, has been actively pursued for decades [1], [2], [3], [4]. As illustrated in Fig. 1a, b, ICF is a process in which frozen deuterium-tritium (DT) nuclear fuel enclosed in an ablator shell is symmetrically compressed by laser-produced thermal X-rays in a gold hohlraum [5,6]. For most ICF processes, the ablator shell is a CH plastic called a glow discharge polymer (GDP) fuel capsule. The compression of X-rays onto the central capsule drives DT fuel and creates a hot spot to achieve ignition implosion [7,8]. In this situation, the created less-dense ablated plasma is pushing against the dense capsule surface. Any perturbations induced by the nonuniformity of the fuel capsule exponentially grow and trigger Rayleigh–Taylor instabilities (Fig. 1c) [9], disrupting the ignition implosion [10]. Therefore, the key to success of implosion experiments lies in the symmetrical compression of nuclear fuel, which largely depends on the uniformity of the GDP fuel capsule (geometric sphericity and surface finish). At present, the degradable mandrel technique is generally employed for the fabrication of GDP fuel capsules, in which millimeter-scale poly-α-methylstyrene (PAMS) HCMs are generally used as templates for GDP fuel capsules (Fig. 1d) [11]: PAMS HCM is first coated with C-H plasma by chemical vapor deposition, forming a GDP/PAMS compound shell; then, the GDP/PAMS compound shell is pyrolyzed to remove the PAMS HCM, and finally, the GDP shell is obtained. It is obvious that the excellent sphericity and surface finish uniformity of PAMS HCMs are the basic guarantees for rigorous geometric uniformity of GDP shells. Therefore, it is important to explore an effective route for the precise fabrication of uniform PAMS HCMs with perfect sphericity and surface finish.

Emerging microencapsulation techniques offer feasible methods for the controlled preparation of uniform HCMs using water-in-oil-in-water (W1/O/W2) compound droplets as precursors [12], [13], [14]. In particular, microfluidics, which enables exquisite fluid manipulation with microchannels, is adopted to generate W1/O/W2 double droplets suspended in the continuous phase [15], [16], [17], [18]. Subsequently, the evaporation of organic solvent from the O phase of W1/O/W2 compound droplets yields solidified microcapsules. It is notable that solidification is a continuous unsteady state and is the key aspect affecting the uniformity of solidified HCM [19]. Unlike colloidal-scale emulsions, which are naturally spherical and dominated by interfacial tension in the continuous phase, the sphericity of compound droplets in millimeter scale is greatly influenced by gravity and buoyancy forces. Therefore, the suspension is generally rotated and tumbled, driven by an external device, generating a rotating shear flow field to randomize these effects [20], [21]. Many factors, including density matching, shear stress exerted by the flow field and interfacial mass transfer, can still influence the spherical uniformity of W1/O/W2 compound droplets during solidification [22], [23]. Great efforts have been made to precisely control the deformation of W1/O/W2 double droplets in a shear flow field during solidification and thus improve the uniformity of leading HCMs in millimeter scale. Researchers at Lawrence Livermore National Laboratory (LLNL) improved the spherical uniformity of HCMs by increasing O-W interfacial tension, lowering flow field shear stress and inhibiting surface defects onside HCMs by adjusting the balance of interfacial osmotic pressure [24], [25], [26]. We previously numerically simulated the deformation dynamics of compound droplets under shear flow and adjusted the density matching between the three phases to eliminate the effects of gravity and buoyancy forces to improve the uniformity of HCMs [27], [28], [29]. These works mainly focused on the points of the flow field and force field to improve the uniformity of HCMs. For interfacial mass transfer, the regulation of molecular diffusion across the O-W2 interface is of great significance for the uniformity of HCMs. Our previous work shows that the inhibited diffusion of organic solvent molecules from the O phase to the continuous phase can greatly improve the spherical uniformity of solidified HCMs [22]. Moreover, our previous theoretical investigation shows that the resisted diffusion of water from the continuous phase into the O phase benefits the surface finish of solidified HCMs [30]. However, most efforts are limited to single-directional regulation of interfacial mass transfer, and the investigations on bidirectional regulation of interfacial mass transfer remain lacking. There remains a great challenge of concurrently improving the spherical uniformity and surface finish of HCMs.

Since peroxide bonds have high electronegativity and easily break to form free radicals, peroxides such as di-*tert*-butyl peroxide (DTBP) are generally used to enhance polymer degradation by increasing the abstraction of hydrogen from the polymer and inducing subsequent chain scission [31], [32]. In addition, Shangguan found that DTBP could improve the sphericity of polymer shells [33]. However, the mechanisms of DTBP affecting the deformation and solidification process of W1/O/W2 compound droplets are seldom discussed. Furthermore, the effects of DTBP on the surface finish of polymer microcapsules and its mechanisms remain unknown. Factoring the effects of DTBP on the sphericity of the polymer shells and the activity characteristics of peroxide bonds in DTBP, we explored the distribution of DTBP in the O-W2 system by molecular dynamics simulations (MD). Simulations show that DTBP tends to distribute at the O-W2 interface, forming an interfacial layer, which would regulate the diffusion of molecules. Herein, this work attempts to improve the sphericity and surface finish of PAMS HCMs concurrently using DTBP to bidirectionally regulate the mass transfer across the O-W2 interface (shown in Fig. 1e). In this work, uniform PAMS HCMs with improved sphericity and surface finish were obtained via mass transfer regulation across the O-W2 interface. Moreover, the mechanisms of DTBP affecting the sphericity and surface finish of PAMS HCMs were experimentally and theoretically investigated. Using these improved PAMS HCMs, GDP fuel capsules meeting the stringent requirements for ICF engineering were prepared, and the quality of which was beyond the NIF standard. The methodology and results of this work may provide valuable guidance for practical high-quality production of eligible GDP fuel capsules used in ICF engineering.

## 2 Materials and methods

### 2.1 Experimental details

#### 2.1.1 Materials

PAMS ($\bar{Mw}$ = 280 kg·mol^−1^, DPI < 1.01) was synthesized by Sichuan University [34] and used as received. Poly(vinyl alcohol) (PVA, $\bar{Mw}$ = 13∼23 kg·mol^−1^, 87%∼89% hydrolyzed) and n-octadecyltrimethoxysilane (OTS) were purchased from Sigma–Aldrich, USA. Fluorobenzene (FB) purchased from Shanghai Aladdin Bio-Chem Technology Co., Ltd. was purified by distilling at 85 °C. DTBP was provided by Shanghai Macklin Company and was used without any further treatment. Trans-2-butene (T_2_B) and tetramethyl silane (TMS) were purchased (Sigma–Aldrich, USA) and used as received. All the chemicals were of reagent grade. Deionized water with a specific resistance of 18.3 MΩ·cm generated from a Millipore-Q water purification device was adopted for the preparation of all aqueous solutions. Glass capillaries with different diameters were obtained from World Precision Instruments Co., Ltd. for preparation of the microfluidic device.

#### 2.1.2 Preparation of millimeter-scale PAMS HCMs

**Fig. 2:**
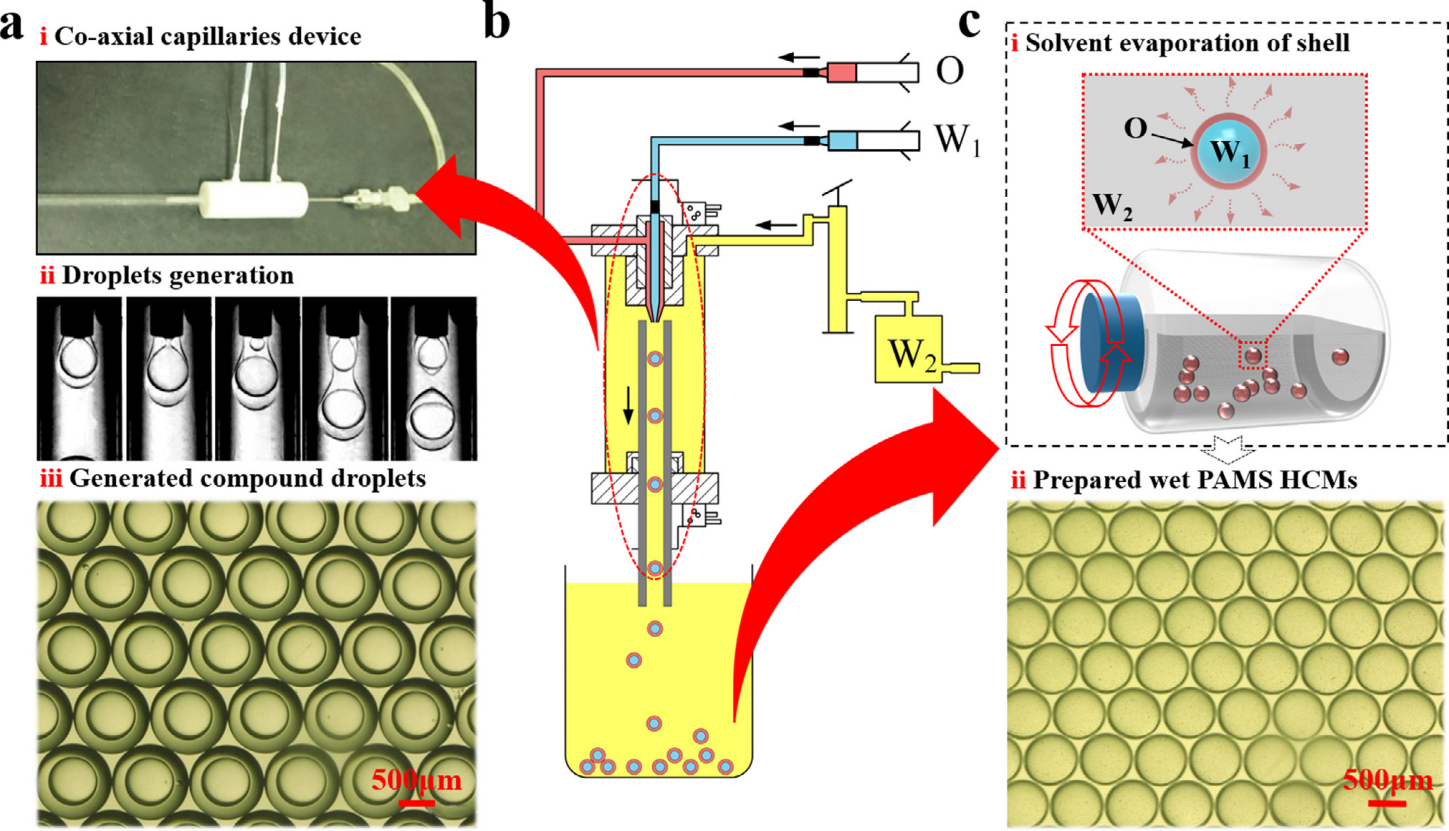
**Schematic of the preparation of HCMs with microencapsulation:** (a) (b) W1/O/W2 compound droplet generation with microfluidics and (c) solvent evaporation-based solidification of W1/O/W2 compound droplets to achieve PAMS HCMs.

**Fig. 3:**
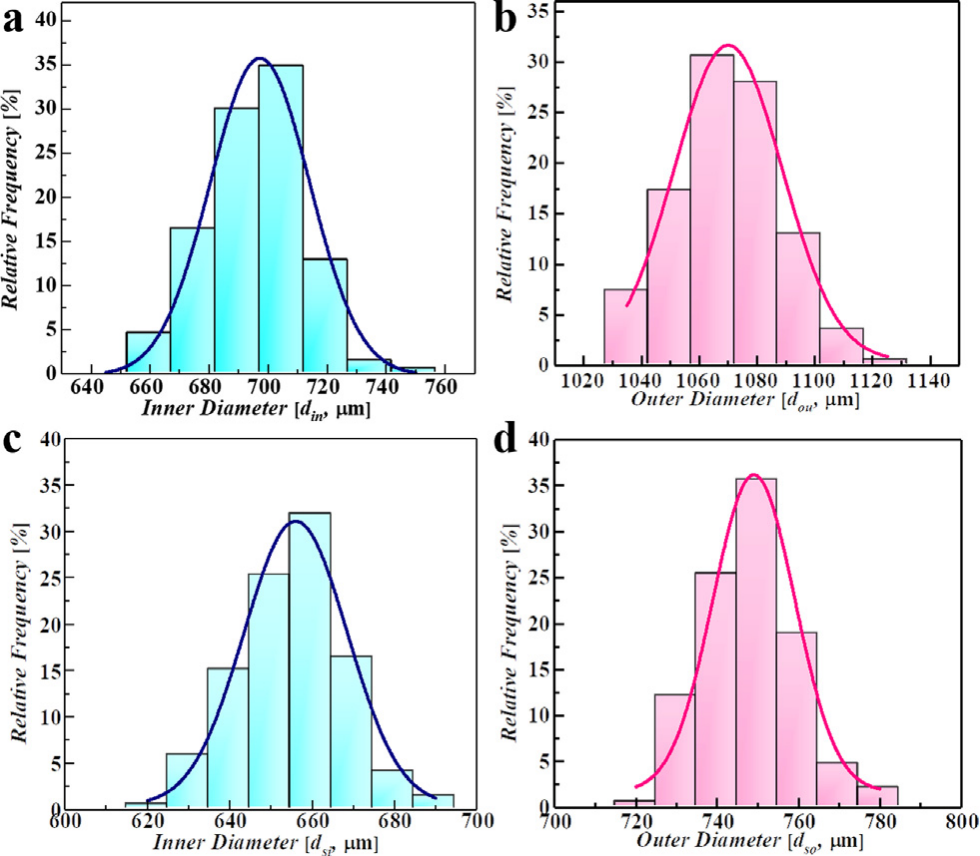
**The size distributions of the W1/O/W2 compound droplets and PAMS HCMs**: (a) The inner diameter and (b) outer diameter distributions of the W1/O/W2 compound droplets; (c) the inner diameter and (d) outer diameter distributions of the solidified PAMS HCMs.

A solvent evaporation-based microencapsulation technique was used to prepare PAMS HCMs on a millimeter scale (Fig. 2). Purified water and 2 wt% PVA aqueous solution were used as the inner and outer phases, respectively. A mixture of 12% PAMS in FB supplemented with various DTBP (0 wt%, 1 wt%, 3 wt%, 5 wt% and 7 wt%) was used as the middle O phase. A co-flow microfluidic device was used to prepare W1/O/W2 compound droplets as precursors. The inner water, middle oil and outer aqueous solutions were separately pumped into the microfluidic channel by three syringe pumps (PHD ULTRA™ Advanced Syringe Pumps, Harvard Apparatus, Inc.) at volumetric flow rates of 0.5 ml/h, 1 ml/h and 200 ml/h, respectively. As illustrated in Fig. 3a, b, the inner (*d_in_*) and outer diameters (*d_ou_*) of the compound droplets in this work are 690 μm and 1060 μm, respectively. The coefficients of variation (CVs) for the sizes of inner and outer drops are 1.7% and 2%, respectively, and are defined as

(1) $$CV=100\%\times {\left(\sum_{i=1}^{n}\frac{{\left(d_{i}-\bar{d}\right)}^{2}}{n-1}\right)}^{\frac{1}{2}}/\bar{d}$$

where *d_i_* is the diameter of the *i*th droplet, *n* is the total number of droplets counted, and $\bar{d}$ is the arithmetic average diameter. All CV values of the inner and outer diameters are less than 3%, indicating that the diameters are coincident with a normal distribution.

The generated compound droplets were collected and suspended in a flask filled with the W2 phase (Fig. 2c). The suspension was then transferred to a thermostatic water bath at 25 °C and rotated driven by an external device at 25 rpm, generating a rotational shear flow field for solidification. With the continuing diffusion of FB, the liquid droplets generally transformed to solid microspheres encapsulating inner water drops. During solidification, all compound droplets were stable and survived. The microspheres encapsulating inner drops were first immersed in ethanol for 48 h. The inner water drop was partly exchanged by ethanol. This process lowers the pressure difference between the inner core with outer surroundings and avoids cracking of the solid microspheres [35]. Subsequently, the inner drop was removed by drying the microspheres at 40 °C, and PAMS HCMs were yielded. As shown in Fig. 3c, d, the inner and outer diameters of the PAMS HCMs are 650 μm and 750 μm, respectively.

#### 2.1.3 Preparation of GDP shells

With the prepared PAMS HCMs, plasma polymerization-based chemical vapor deposition (CVD) was adopted to fabricate the GDP/PAMS compound capsules. In this process, the H_2_, T_2_B and TMS mixture was broken down by high-pressure ionization into inductively coupled plasma, which was uniformly coated on the outside of the PAMS HCMs. In particular, the base pressure in the reactor was controlled to be less than 10^−4^ Pa with a molecular pump and a mechanical pump. The H_2_ and T_2_B mixture was discharged under a pressure condition of 10 Pa and a radio frequency condition of 15 W. The flows of H_2_, T_2_B and TMS were controlled by a mass controller and set at 10 cm^3^/min, 0.4 cm^3^/min and 0.25 cm^3^/min, respectively. The GDP/PAMS compound capsules with a GDP thickness of 60 μm were prepared in a controlled manner and were then pyrolyzed at 305 °C for 40 h to remove the PAMS HCMs. The GDP capsules were finally obtained.

#### 2.1.4 Characterization

The optical microscope images of W1/O/W2 compound droplets, PAMS HCMs and the diameters were obtained by digital microscopy (VXH Keyence, Japan). The surface finish of the HCMs was characterized by white light interference (WLI) microscopy (WYKD-NT1100) in phase-shift interferometry (PSI) mode. The WLI characterization area was 90 μm × 120 μm. Three areas of a PAMS HCM were measured, and the average surface roughness (*Rt*) value was statistically calculated. The spherical uniformity of the PAMS HCM was characterized by out-of-round (*δ_OOR_*) with X-ray radiography. Eight HCM radii in different directions were measured, and maximum (*R*_max_) and minimum (*R*_min_) radii could be obtained. The measurement method was introduced in our previous work in detail [22]. The *δ_OOR_* is defined as

(2) $$\delta_{OOR}=R_{\max}-R_{\min}$$

This equation shows that a decreasing *δ_OOR_* value indicates increasing sphericity of the PAMS HCM. In this experiment, thirty HCMs in each batch were randomly sampled, measured and statistically calculated to obtain the cumulative *δ_OOR_* frequencies.

### 2.2 Molecular dynamics simulations

The oil layer width of a millimeter-scale compound droplet ranges over several hundred micrometers. Such a membrane is wide enough to be described with a continuum model. To derive the thermodynamics and transportation properties, including diffusivity, solubility and interfacial mass transfer, a microscopic O-W2 interface at the nanoscale was constructed and studied by molecular simulations. Since the essence of O-W2 interfacial mass transfer was the diffusion of FB through the oil phase to the continuous phase, the PAMS-FB-DTBP/H_2_O interface in the simulation was simplified as an FB-DTBP/H_2_O interface. Two O-W2 interfaces, including the FB/H_2_O interface and FB-DTBP/H_2_O interface, were simulated by MD. The size of the two interface systems fluctuated at approximately 15 nm × 10 nm × 10 nm. The FB/H_2_O interface system contained 33332 H_2_O molecules and 3090 FB molecules. The FB-DTBP/H_2_O interface system contained 33,332 H_2_O molecules, 2,910 FB molecules and 100 DTBP molecules, constructing an oil phase with 5% DTBP dissolved in FB. The present molecular simulations for FB and DTBP molecules were performed using the GAFF small molecule force field [36]. The TIP3P model was adopted for H_2_O molecules. The intermolecular interactions were described by RESP charges [37]. With this physical model and force field, the thermodynamics and transportation properties, including diffusivity, solubility and interfacial mass transfer behavior, were investigated with OpenMM [38].

**Fig. 4:**
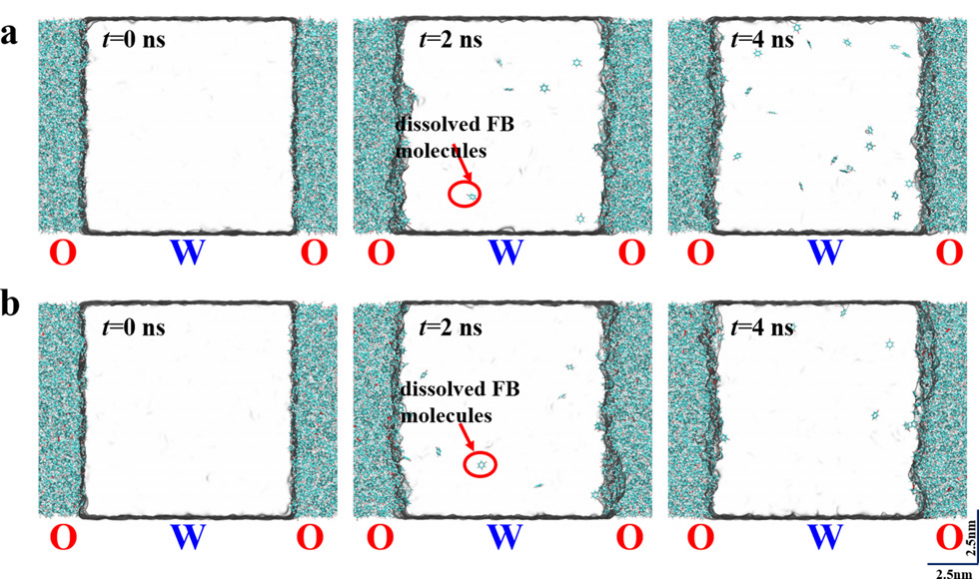
**Snapshots of the dissolving process of FB in the W2 phase at different system interfaces:** (a) FB/H_2_O system interface and (b) FB-DTBP/H_2_O system interface. The scale in both directions is 2.5 nm.

**Fig. 5:**
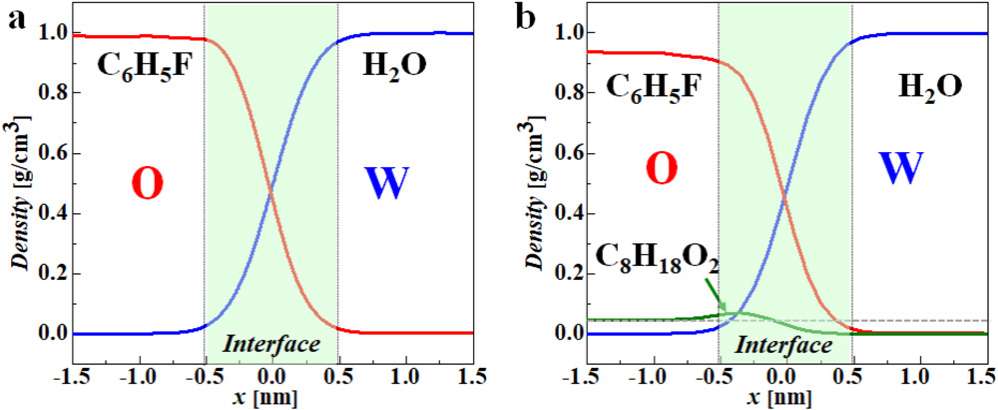
**Density distributions for various components at O-W2 interfaces:** (a) FB-H_2_O system interface and (b) FB-DTBP/H_2_O system interface.

With the above molecular dynamic simulations, the effects of DTBP in the oil phase on the solidification of W1/O/W2 compound droplets were explored. In this work, the two systems were first balanced in the NVT ensemble for 5 ns at a temperature of 298 K controlled by the Langevin thermostat. The initial interfacial (see *t* = 0 ns in Fig. 4) components were investigated by calculating the density distributions of the components near the O-W2 interface. As shown in Fig. 5, the width of the initial O-W2 interface ranging from -0.5∼0.5 nm is 1 nm. Compared with the FB-H_2_O system interface, the addition of DTBP changes little for the FB-DTBP/H_2_O system interface. From the density distribution of DTBP in the FB-DTBP/H_2_O system interface (Fig. 5b), the density of DTBP at the FB-DTBP/H_2_O interface is higher than that in the bulk phase, which indicates that DTBP molecules tend to accumulate at the O-W2 interface.

**Fig. 6:**
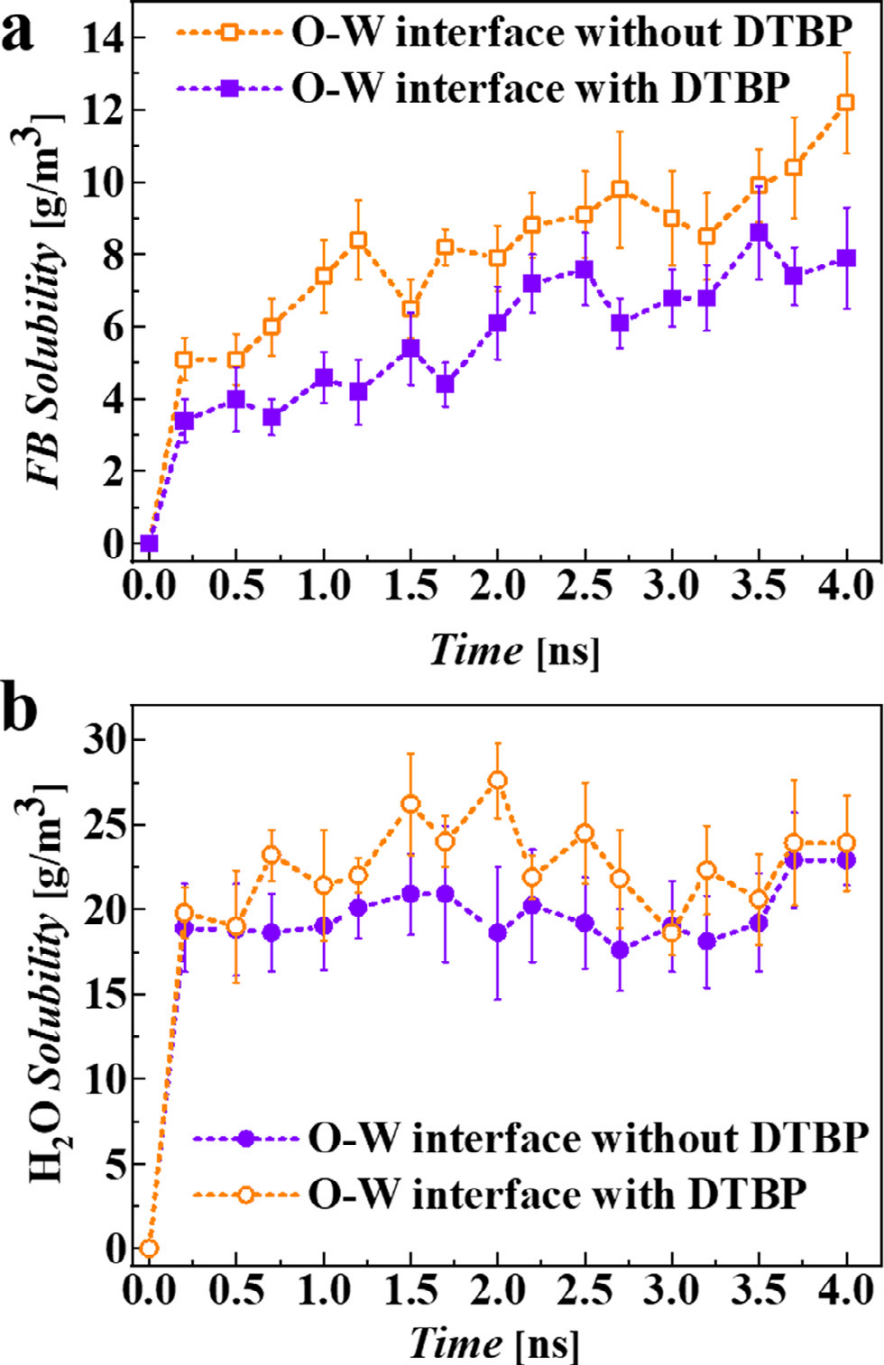
**Dissolving curves of FB and H_2_O molecules at different system interfaces:** (a) FB/H_2_O system interface and (b) FB-DTBP/H_2_O system interface.

The dissolving processes of FB in the two systems were then simulated by controlling the temperature at 298 K with a Nose–Hoover thermostat. The simulation for the dissolving process lasted 4 ns. Moreover, we quantitatively investigated the dissolving process by calculating the number of diffused FB molecules in the W2 bulk region and the number of H_2_O molecules dissolved in the O region. For each system, the simulation was repeated 8 times, and the average values were calculated. The number of molecules diffusing across the O-W2 interface increases from zero to a saturated value as the mass transfer process proceeds. As illustrated in Fig. 4 and Fig. 6a, comparing the dissolving behaviors of FB molecules between the two systems, the diffused FB molecules in the W2 phase in the FB-DTBP/H_2_O system are fewer than those in the FB/H_2_O system at the same time, which indicates that the addition of DTBP in the O phase can effectively inhibit the diffusion process of FB molecules. Moreover, the simulation results also show that the addition of DTBP to the O phase can also inhibit the diffusion of H_2_O into the O region in a certain degree (Fig. 6b). The simulation results indicate that the O-W2 interfacial mass transfer is bidirectionally inhibited by DTBP.

## 3 Results and discussion

**Fig. 7:**
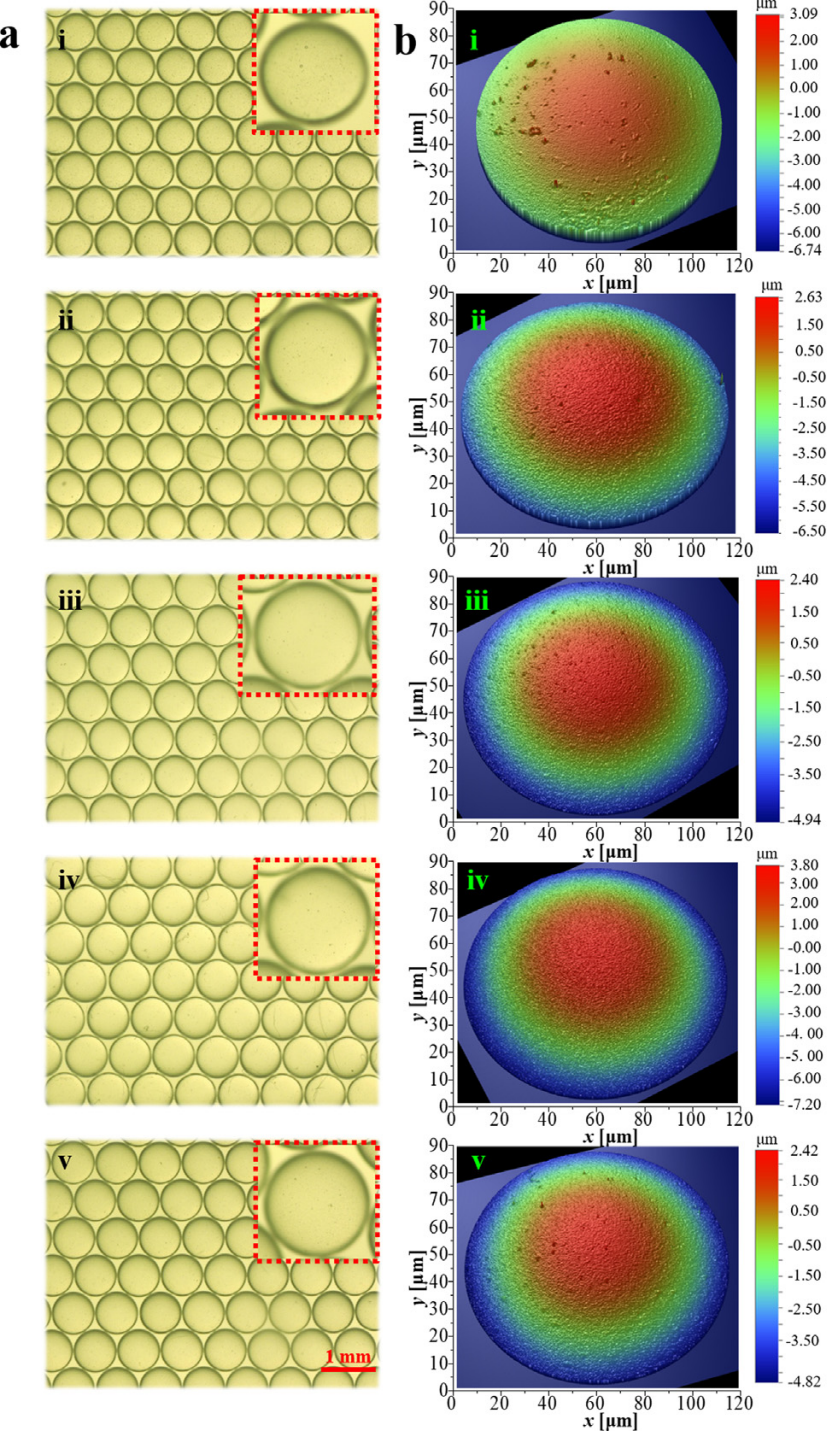
**Morphologies of the PAMS HCMs:** (a) optical microphotographs and (b) white light interference images of the PAMS HCMs prepared with DTBP at various mass fractions: (i) 0%, (ii) 1%, (iii) 3%, (iv) 5% and (v) 7%.

**Fig. 8:**
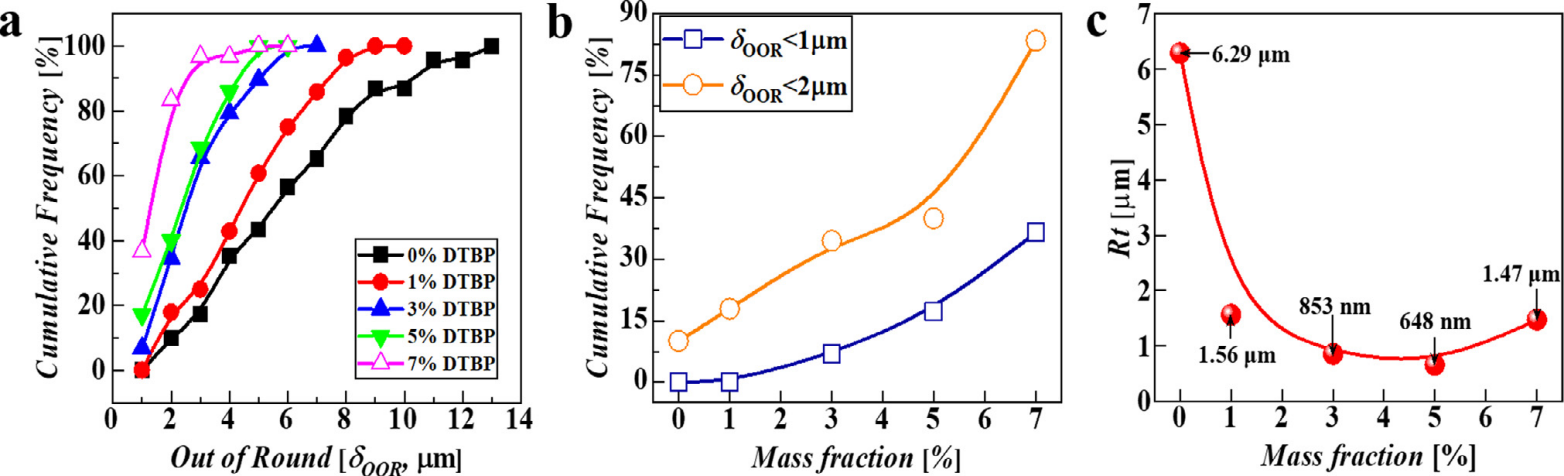
**Quantitative statistical characterizations of the sphericity and surface finish of PAMS HCMs**: (a) (b) effects of the DTBP mass fraction on the sphericity and (c) surface finish of PAMS HCMs.

Using the present mass-transfer-inhibited interface, highly spherical and surface uniform PAMS HCMs can be controlledly fabricated. Fig. 7 illustrates the optical microphotographs and WLI images of the controlledly prepared PAMS HCMs. Compared with PAMS HCMs prepared without DTBP (Fig. 7a i), the optical micrographs of PAMS HCMs prepared with DTBP in Fig. 7a ii-v demonstrate high spherical uniformity. The magnified images in the insets of Fig. 7a show that the surface finish of the PAMS HCMs prepared with DTBP is smoother than that prepared without DTBP. The WLI morphologies of PAMS HCMs verify this result. According to the above characterization results, the effects of DTBP mass fraction on the statistical distributions of *δ_OOR_* and *R_t_* are included in Fig. 8a-c, respectively. As shown in Fig. 7a, b, the addition of DTBP can effectively improve the sphericity of PAMS HCMs. Specifically, the cumulative frequency profile increases with the increasing mass fraction of DTBP added to the O phase (Fig. 8a). To show these effects more intuitively, the fractions of HCMs with *δ_OOR_* less than a certain value (1 μm and 2 μm) are summarized in Fig. 8b. Specifically, the fractions of PAMS HCMs with *δ_OOR_* < 1 μm and *δ_OOR_* < 2 μm increase with an increase in the DTBP mass fraction. For example, when no DTBP (0% DTBP) is added to the O phase, the fractions of PAMS HCMs with *δ_OOR_* < 1 μm and *δ_OOR_* < 2 μm are approximately 0% and 10%, respectively. However, when the mass fraction of DTBP is 7%, the fractions of PAMS HCMs with *δ_OOR_* < 1 μm and *δ_OOR_* < 2 μm are approximately 40% and 85%, respectively. In addition, as illustrated in Fig. 8c, the surface roughness *R_t_* of PAMS HCMs is also statistically calculated. The addition of DTBP greatly decreases the surface roughness of PAMS HCMs from 6 μm to several hundred nanometers. These quantitative statistical results verify the optical and WLI morphologies of PAMS HCMs in Fig. 7.

During solidification, multiple fluid flows and mass transfer exert combined effects on the uniformity of solidified PAMS HCMs. In this work, the physical conditions of multiple fluid flows are kept the same for each sample. Therefore, the mass transfer across the O-W2 interface is considered the only process affecting the uniformity of HCMs. From the above simulations, the addition of DTBP into the O phase can effectively inhibit the diffusion of FB and H_2_O molecules. With this inhibited-mass-transfer interface, the solidification of W1/O/W2 compound droplets would be extended. The solidification of W1/O/W2 compound droplets prepared with various concentrations of DTBP was studied by recording the changing diameters of compound droplets. Using the changing diameters, the changes in the concentration (*c*_t_) and density (*ρ*_t_) of the O phase at different times can be deduced:

(3) $$c_{t}=\frac{\rho_{o}\cdot c_{o}}{\rho_{o}-\left(\frac{R_{o}^{3}-R_{t}^{3}}{R_{o}^{3}-r_{o}^{3}}\right)\cdot \rho_{FB}}\times 100\%$$

(4) $$\rho_{t}=\frac{\left(R_{o}^{3}-r_{o}^{3}\right)\cdot \rho_{t}-\left(R_{o}^{3}-R_{t}^{3}\right)\cdot \rho_{FB}}{\left(R_{t}^{3}-r_{o}^{3}\right)}$$

where *ρ*_o_ and *c*_o_ are the initial density and PAMS concentration of the O phase, respectively. *Ρ*_FB_ is the density of purified FB solvent. *R*_o_ and *R*_t_ are the initial and changing outer radii of the W1/O/W2 compound droplet, respectively. *R*_o_ is the inner radius of the W1/O/W2 compound droplet, which is considered unchanged during solidification.

**Fig. 9:**
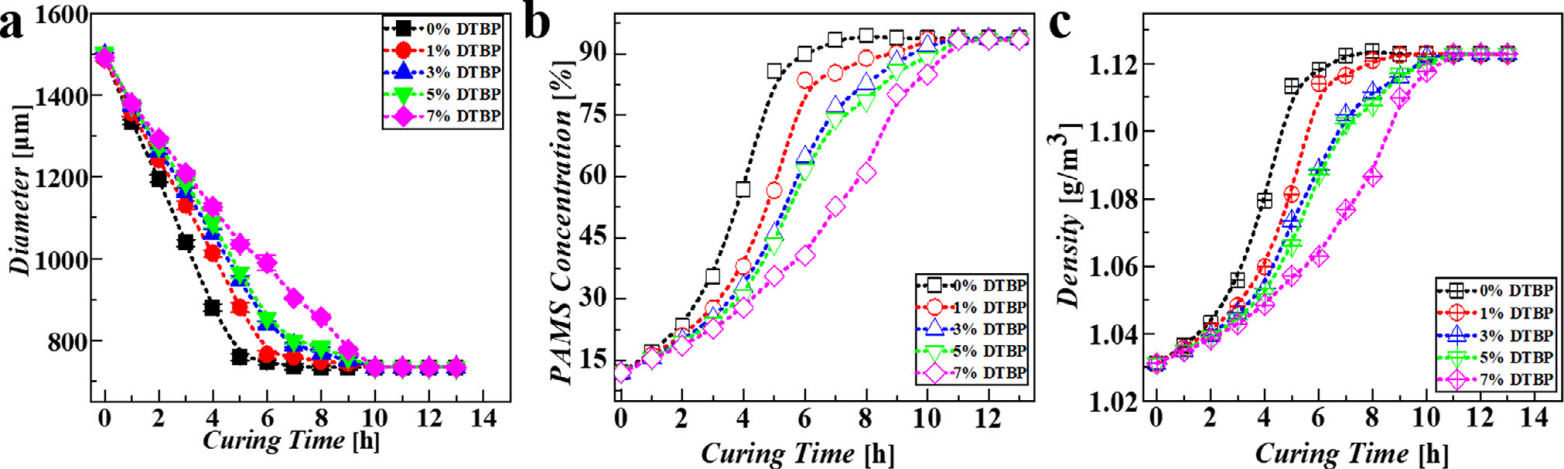
**Effects of DTBP on the solidification of W1/O/W2 compound droplets:** effects of the DTBP mass fraction on the changing (a) diameters, (b) O phase concentrations and (c) densities of W1/O/W2 compound droplets.

With these experiments, the effects of the DTBP mass fractions on the solidification of W1/O/W2 compound droplets are shown in in Fig. 9. As illustrated in Fig. 9a, the droplet shrinks and the diameter decreases with the outward diffusion of FB. The diameter of compound droplet reaches a constant, which signals the end of solidification. Moreover, with increasing the concentration of DTBP, the decreasing rate of the droplet size decreases. This is because the addition of DTBP can inhibit the diffusion of FB from the O phase. Based on the changing size of W1/O/W2 compound droplet, the relevant profiles of O phase changing concentration and density can be obtained (Fig. 9b, c). As illustrated in Fig. 9b, the PAMS concentration increases slowly at first and then rapidly. Correspondingly, the density of the O phase also increases as solidification proceeds. Combining the numerical simulations with this experimental research, we found that DTBP could inhibit the diffusion of FB and extend the solidification of W1/O/W2 compound droplets.

During solidification, restoring forces, such as interfacial tension, can only work when the droplets remain in the liquid state and drive the droplet to resist deformation. In previous work, a percolation zone was defined to divide the liquid and solid states of the W1/O/W2 compound droplets during solidification. Moreover, the extension of solidification can postpone the appearance of the percolation zone, which can effectively prolong the W1/O/W2 compound droplets staying in the liquid state [22]. Therefore, the restoring forces can work adequately to maintain spherical uniformity until the percolation zone is reached. In addition, previous work also shows that inhibited interfacial mass transfer can eliminate the interfacial Marangoni convection to a certain degree, which benefits the surface finish of solidified HCMs [39]. Moreover, the H_2_O molecules in the W2 phase diffused and dissolved into the O phase, which is another origin of surface defects. Specifically, the dissolved H_2_O molecules would nucleate and form small water drops adhering to the polymer molecules during solidification. The evaporation of these small drops when the O layer is completely solidified triggers surface defects. Simulation results (Fig. 6b) show that H_2_O molecules migrating toward the O phase gradually reach equilibrium. More importantly, the addition of DTBP to the O phase can resist the migration of H_2_O molecules toward the O membrane layer. Therefore, the surface finish of solidified PAMS HCMs can be greatly improved. Above all, the addition of DTBP to the O phase can effectively inhibit the mass transfer bidirectionally, thus improving the surface and spherical uniformity of the solidified PAMS HCMs.

**Fig. 10:**
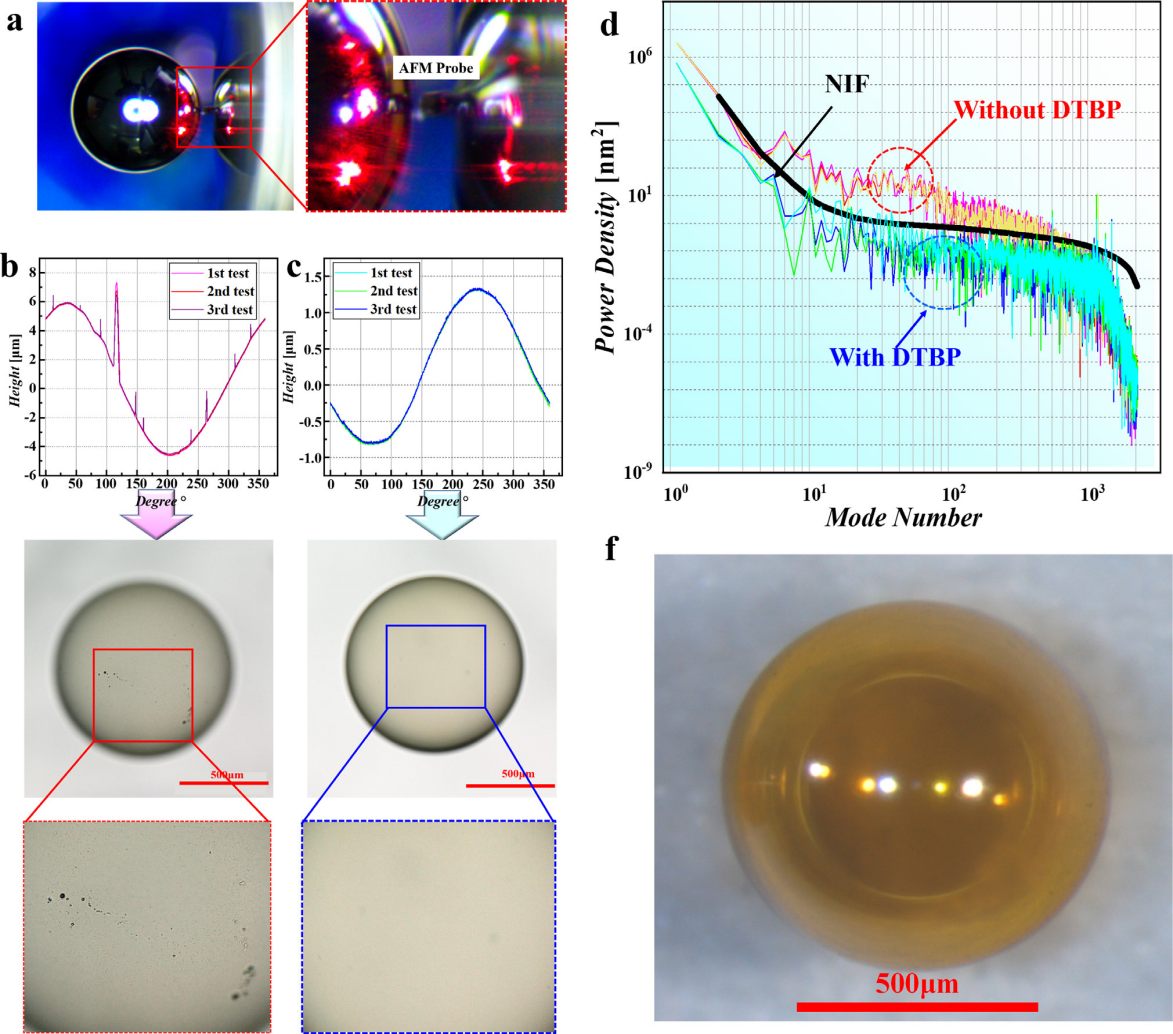
**Characterizations of GDP fuel capsules with AFM and optical microscope:** (a) AFM measurement of GDP capsule under auxiliary video surveillance; (b) outer traces and optical morphology of a GDP capsule prepared with PAMS HCM; (c) outer traces and optical morphology of a GDP capsule prepared with PAMS/DTBP HCM; (d) calculated power spectra of GDP capsules and (f) 3-D optical morphology of a GDP capsule with improved qualities.

Adjusting for the effects of DTBP on the surface finish, sphericity and solidification rate, PAMS HCMs with 5% DTBP were used for preparation of GDP capsules. The GDP/PAMS compound capsules were prepared with the method introduced in Section 2.1.3. The compound capsules were then pyrolyzed at 305 °C to remove the inner PAMS HCMs, obtaining GDP capsules. The 1D mode-power spectrum density (PSD) curve based on atomic force microscopy (AFM) is generally used to characterize the sphericity and surface finish of GDP capsules in ICF (shown in Fig. 10a). The outer traces around the GDP capsules were first obtained using AFM. Compared with the outer traces of the GDP capsules prepared with PAMS/DTBP HCMs (Fig. 10c), obvious perturbations are observed on the GDP capsules prepared with PAMS HCMs (without DTBP). This result indicates that the outer surface of GDP capsules is greatly improved with PAMS HCMs in the presence of DTBP. This is verified by the optical morphologies shown in Fig. 10b, c. Obvious defects are observed on the GDP capsules prepared with PAMS HCMs (without DTBP). The outer traces of surface height were transferred into a 1D power spectrum by Fourier fast transition (FFT) (details in Appendix) [40]. In the mode-PSD curve, low modes 2–6 are used to characterize the sphericity, while mid modes 7–25 are employed to characterize the outer surface qualities of the GDP capsules. As illustrated in Fig. 10d, a national ignition facility (NIF) standard curve is used to judge whether the GDP capsule meets the requirements. Specifically, the GDP capsule meets these requirements when the value of the actual curve of the GDP capsule is lower than that of the NIF standard curve in all mode sections. Fig. 10d shows that the PSD curves of the GDP capsule prepared with PAMS/DTBP HCM are lower than the NIF curve, while those of the GDP capsule prepared with PAMS HCM (without DTBP) are much higher. This result indicates that the sphericity and outer surface finish of the GDP capsule are improved with the PAMS/DTBP HCMs.

## 4 Conclusion

In this work, millimeter-scale PAMS HCMs were fabricated by a solvent evaporation-based microencapsulation technique. Monodisperse W1/O/W2 compound droplets used as precursors for PAMS HCMs were prepared with a two-step microfluidic device. A third additive, DTBP, was introduced in the O phase of W1/O/W2 compound droplets to explore possible effects on concurrently improving the sphericity and surface finish uniformity of millimeter-scale PAMS HCMs. The experimental results show that the addition of DTBP can greatly improve both the sphericity and surface finish of PAMS HCMs. Specifically, the frequency of PAMS HCMs with an *δ_OOR_* less than 1 μm increases by approximately 40%, and the maximum outer surface roughness decreases from 6 μm to 0.6 μm. In addition, the effects of adding DTBP were clarified by combining experiments and molecular dynamic simulations. DTBP molecules tend to distribute at the O-W2 interface, forming an interfacial layer, which effectively inhibits the diffusion of H_2_O molecules toward the O phase and FB molecules toward the W2 phase. The inhibited diffusion of H_2_O molecules can greatly decrease the surface defects outside the PAMS HCM surface. In addition, the inhibited diffusion of FB molecules to the W2 phase can resist the mass transfer process and extend the solidification of W1/O/W2 compound droplets, which is also verified by experiments. The extended solidification will prolong the duration of the droplets in the liquid state, which facilitates sufficient action of the restoring forces for maintaining the sphericity of W1/O/W2 droplets and the consequent solidified HCMs. Finally, using these PAMS HCMs with concurrently improved spherical uniformity and surface finish, GDP fuel capsules beyond the NIF standard for ICF engineering were prepared. The methodology of this work may provide valuable guidance for practical high-quality production of eligible GDP fuel capsules used in ICF engineering.

## Declaration of competing interest

The authors declare that they have no conflicts of interest in this work.

## Appendix

**FFT based 1D power spectrum calculation**

Generally, the complete outer trace obtained by measuring the outline of the GDP capsule can be considered a 1D discrete time sequence. Supposing the signal is stable, the mixed information composed of periodic and random components is characterized in Eq. A1:

(A1) $$x\left(t\right)=a_{0}+\sum_{j=1}^{p}\varepsilon_{j}\cos\left(2\pi f_{j}t-\varphi_{0j}\right)+r\left(t\right)$$

where *a*_0_ is the direct current (DC) component, *ε_j_, f_j_*, and *φ_0j_* are the amplitude, frequency and initial phase of the *j*_th_ harmonic, respectively. In addition, *r*(*t*) is the random component. Taking N points in the measured signal of the complete circumference, these points are expressed as *x*(*n*) (*n* = 1, 2, ….N). Assuming the sampling time interval is T = 1, the fundamental frequency is *ω*_0_ = 2π/*N*. Then, the discrete FFT of the signal can be characterized as Eq. A2:

(A2) $$X\left(m\omega_{0}\right)=\sum_{n=0}^{N-1}x\left(n\right)e^{-2\pi imn/N}$$

For a complete circumferential measurement sequence, *ω*_0_ indicates the first harmonic and *mω*_0_ indicates the harmonic completes *m* cycles. So *m* is the mode. In this case, ∥X(m)∥ is the amplitude of the *m*_th_ harmonic component. Taking the mode (*m*) as the independent variable and the power of the amplitude of the harmonic component as the dependent variable, the FFT-based 1D mode-power spectrum (*P*_1_*_D_* (*m*)) is

(A3) $$P_{1D}\left(m\right)=2\times {\left(\frac{1}{N}∥X(m)∥\right)}^{2}$$

Based on the sampling theorem, the harmonic components with mode *m* = 0,1,2... N/2 can be distinguished at most in the N-point data sequence. Therefore, the calculation result of the FFT is a conjugate symmetric structure of real and imaginary centered on the zero order (DC component). With the above equations, *P*_1_*_D_* (*m*) can be expressed by the *m*_th_ harmonic amplitude (*ε_m_*) as

(A4) $$P_{1D}\left(m\right)=\frac{\varepsilon_{m}^{2}}{2}$$

## References

[1] Li H.Y., Huang Y.B., Jiang S.E. (2015). A unified modeling approach for physical experiment design and optimization in laser driven inertial confinement fusion. Fusion Eng. Des..

[2] Glenzer S.H., MacGowan B.J., Michel P. (2010). Symmetric inertial confinement fusion implosions at ultra-high laser energies. Science.

[3] Lindl J., Landen O., Edwards J. (2014). Review of the National Ignition Campaign 2009-2012. Phys. Plasmas.

[4] Hua S.X., Goncharov V.N., Radha P.B. (2019). Microphysics studies for direct-drive inertial confinement fusion. Nucl. Fusion.

[5] A.Hurricane O., Callahan D.A., Casey D.T. (2014). Fuel gain exceeding unity in an inertial confined fusion implosion. Nature.

[6] Jiang S.E., Wang F., Ding Y.K. (2019). Experimental progress of inertial confinement fusion based at the ShenGuang-III laser facility in China. Nucl. Fusion.

[7] Zhang F., Cai H.B., Zhou W.M. (2020). Enhanced energy coupling for indirect-drive fast-ignition fusion targets. Nat. Phys..

[8] Kline J.L., Batha S.H., Benedetti L.R. (2019). Progress of indirect drive inertial confinement fusion in the United States. Nucl. Fusion.

[9] Betti R., Hurricane O.A. (2016). Inertial-Confinement fusion with lasers. Nat. Phys..

[10] Gatu Johnson M., Appelbe B.D., Chittenden J.P. (2018). Impact of asymmetries on fuel performance in inertial confinement fusion. Phys. Rev. E.

[11] Du K., Liu M.F., Wang T. (2018). Recent progress in ICF target fabrication at RCLF. Matter Radiat. Extrem..

[12] Shang L.R., Cheng Y., Wang J. (2014). Double emulsions from a capillary array injection microfluidic device. Lab Chip.

[13] Shang L.R., Cheng Y., Zhao Y.J. (2017). Emerging droplet microfluidics. Chem. Rev..

[14] Wang J., Shao C.M., Wang Y.T. (2020). Microfluidics for medical additive manufacturing. Engineering.

[15] Christopher G.F., Anna S.L. (2007). Microfluidic methods for generating continuous droplet streams. J. Phys. D Appl. Phys..

[16] Liu H.H., Zhang Y.H. (2011). Droplet formation in microfluidic cross-junctions. Phys. Fluids.

[17] Che Z.Z., Yap Y.F., Wang T.Y. (2018). Flow structure of compound droplets moving in microchannels. Phys. Fluids.

[18] Liu F., Yin K., Liu H.H. (2021). A microfluidic synthesis method for preparation and regulation of 3-aminophenol formaldehyde resin spheres. React. Funct. Polym.

[19] Bhandarkar S., Paguio R., Elsner F. (2016). Understanding the critical parameters of the PAMS Mandrel fabrication process. Fusion Sci. Technol..

[20] Fu T.T., Wu Y.N., Ma Y.G. (2012). Droplet formation and breakup dynamics in microfluidic flow-focusing devices: From dripping to jetting. Chem. Eng. Sci..

[21] Wang J.T., Wang X.Y., Tai M. (2016). Oriented shift and inverse of the daughter droplet due to the asymmetry of grand-daughter droplets of multiple emulsions in a symmetric flow field. Appl. Phys. Lett..

[22] Chen Q., Pan D.W., Qi X.B. (2019). Controlled fabrication of solid-shelled capsules with designed geometry sphericity. Chem. Eng. Sci..

[23] Luo Z.Y., Bai B.F. (2016). Dynamics of nonspherical compound capsules in simple shear flow. Phys. Fluids.

[24] Mcquillan B.W., Greenwood A. (1999). Microencapsulation process factors which influence the sphericity of 1 mm o.d. poly(α-methylstyrene) shells for ICF. Fusion Sci. Technol..

[25] Mcquillan B.W., Eisner F.H., Stephens R.B. (1999). The use of CaCl2 and other salts to improve surface finish and eliminate vacuoles in ICF microencapsulated shells. Fusion Sci. Technol..

[26] Cook R.C., Gresho P.M., Hamilton K.E. (1998). Examination of some droplet deformation forces related to NIF capsule sphericity. J. Moscow Phys. Soc..

[27] Liu M.F., Chen S.F., Qi X.B. (2014). Improvement of wall thickness uniformity of thick-walled polystyrene shells by density matching. Chem. Eng. J..

[28] Chen Y.P., Liu X.D., Zhang C.B. (2015). Enhancing and suppressing effects of an inner droplet on deformation of a double emulsion droplet under shear. Lab Chip.

[29] Chen Y.P., Liu X.D., Shi M.H. (2013). Hydrodynamics of double emulsion droplet in shear flow. Appl. Phys. Lett..

[30] Zhou B., Pei C., Chen Y.P. (2019). Interfacial mass transfer of water for fluorobenzene/aqueous solution system in double emulsion. Int. J. Heat Mass Tran..

[31] Ma S., Huang Y.W., Li F. (2015). Effects of radical initiator on the thermal degradation of poly(alpha-methylstyrene). Polym. Mater. Sci. Eng..

[32] Sterling W.J., Kim Y.C., McCoy B.J. (2001). Peroxide enhancement of poly(α-methylstyrene) thermal degradation. Ind. Eng. Chem. Res..

[33] X.Y. Shangguan, Effects of DTBP and other factors on the thermal degradation performance of poly(alpha-methylstyrene) [D], 2017.

[34] Moreau L., Levassort C., Blondel B. (2009). Recent advances in development of materials for laser target. Laser Part Beams.

[35] Pan D.W., Liu M.F., Chen Q. (2018). Investigation of craze and cracks of polystyrene shells during drying process. Fusion Sci. Technol..

[36] Wang J., Wolf R.M., Caldwell J.W. (2004). Development and testing of a general AMBER force field. J. Comput. Chem..

[37] Tian L., Chen F.W. (2012). Multiwfn: A multifunctional wavefunction analyzer. J. Comput. Chem..

[38] Peter E., Jason S., Chodera J.D. (2017). OpenMM 7: Rapid development of high performance algorithms for molecular dynamics. Plos Comput. Biol..

[39] Subramanian P., Zebib A., McQuillan B.W. (2005). Axisymmetric Marangoni convection in microencapsulation. Acta Astronautica.

[40] Zhao X.S., Sun T., Gao D.Z. (2005). Measurement and characteristic evaluation of target circumferential topography with AFM. High Power Laser and Particle Beams.

